# A platform for oncogenomic reporting and interpretation

**DOI:** 10.1038/s41467-022-28348-y

**Published:** 2022-02-09

**Authors:** Caralyn Reisle, Laura M. Williamson, Erin Pleasance, Anna Davies, Brayden Pellegrini, Dustin W. Bleile, Karen L. Mungall, Eric Chuah, Martin R. Jones, Yussanne Ma, Eleanor Lewis, Isaac Beckie, David Pham, Raphael Matiello Pletz, Amir Muhammadzadeh, Brandon M. Pierce, Jacky Li, Ross Stevenson, Hansen Wong, Lance Bailey, Abbey Reisle, Matthew Douglas, Melika Bonakdar, Jessica M. T. Nelson, Cameron J. Grisdale, Martin Krzywinski, Ana Fisic, Teresa Mitchell, Daniel J. Renouf, Stephen Yip, Janessa Laskin, Marco A. Marra, Steven J. M. Jones

**Affiliations:** 1 Canada’s Michael Smith Genome Sciences Centre, Vancouver, BC Canada; 2 Bioinformatics Graduate Program, Faculty of Science, University of British Columbia, Vancouver, BC Canada; 3 Department of Medical Oncology, BC Cancer, Vancouver, BC Canada; 4 Pancreas Centre BC, Vancouver, BC Canada; 5 Department of Pathology and Laboratory Medicine, Faculty of Medicine, University of British Columbia, Vancouver, BC Canada; 6 Department of Medical Genetics, University of British Columbia, Vancouver, BC Canada; 7 Department of Molecular Biology and Biochemistry, Simon Fraser University, Burnaby, BC Canada

**Keywords:** Genetics research, Cancer genomics, Computational platforms and environments, Data integration, Cancer genetics

## Abstract

Manual interpretation of variants remains rate limiting in precision oncology. The increasing scale and complexity of molecular data generated from comprehensive sequencing of cancer samples requires advanced interpretative platforms as precision oncology expands beyond individual patients to entire populations. To address this unmet need, we introduce a Platform for Oncogenomic Reporting and Interpretation (PORI), comprising an analytic framework that facilitates the interpretation and reporting of somatic variants in cancer. PORI integrates reporting and graph knowledge base tools combined with support for manual curation at the reporting stage. PORI represents an open-source platform alternative to commercial reporting solutions suitable for comprehensive genomic data sets in precision oncology. We demonstrate the utility of PORI by matching 9,961 pan-cancer genome atlas tumours to the graph knowledge base, calculating therapeutically informative alterations, and making available reports describing select individual samples.

## Abstract (web summary)

The interpretation of somatic variants in cancer is challenging due to the scale and complexity of sequencing data. Here, the authors present PORI, an open-source framework for interpreting somatic variants in cancer using graph knowledge base tools, automated reporting, and manual curation.

## Introduction

As the research and clinical applications of human cancer sequencing for precision medicine grow, there is an increased demand for the interpretation and reporting of genomic data in both research and clinical settings. Automation of cancer analysis research pipelines has improved the speed of reporting and the reproducibility of results. However, portions of the analysis remain refractory to automation. The human interpretation of genomic data remains one of the largest bottlenecks in comprehensive precision oncology^1,2^.

To address this problem, a number of cancer knowledge bases have been created, including: OncoKB^3^; Clinical Interpretation of Variants in Cancer (CIViC)^4^; Cancer Genome Interpreter (CGI)^5^; Catalogue of Somatic Mutations in Cancer (COSMIC)^6^; Jackson Laboratory Clinical Knowledge Base (JAX-CKB)^7^; Precision Medicine Knowledge Base (PMKB)^8^; My Cancer Genome^9^; Personalized Cancer Therapy (PCT)^10^; and Cancer Driver Log (CanDL)^11^. Despite the increasing availability of publicly accessible knowledge bases, these resources are distributed across a broad landscape of clinical and biological knowledge that is often disjointed and of varying structure. Integration of these tools into a reporting workflow to improve coverage^12^ is essential, yet left largely to individual users.

The increasing scale and complexity of the genomic and clinical data collected for sequenced tumour samples requires flexible analytic platforms suitable for automation^2^, both for the annotation of molecular profiles as well as the concise reporting of such information. While there are visualization tools^13,14^ and commercial reporting applications available^7,15,16^, there are few open-source reporting alternatives^17,18^. Despite previous work demonstrating improvements in clinical comprehension of complex genomic data using interactive over static reports^19^, there are currently no open-source web applications for reporting in precision oncology. Open-source software is essential for promoting reproducibility and transparency in both research and healthcare, allowing the community to evaluate the softwares implementations and ensure their correctness^20^. This is particularly important in research where the outcomes and insights will ultimately impact patient care. Furthermore, there is limited ability to build on and learn from closed source implementations within the research and clinical communities^21^.

While institutions have aimed to standardize workflows with respect to laboratory methods or even the bioinformatic tools used in variant calling^22^, reporting and annotation workflows remain diverse^15^. Here we present a fully open-source research platform that integrates variant annotation through knowledge base matching into a precision oncology workflow and provides users a reporting interface to curate, edit, and interact with the resulting data.

## Results

### Flexible open source reporting with PORI

**Fig. 1:**
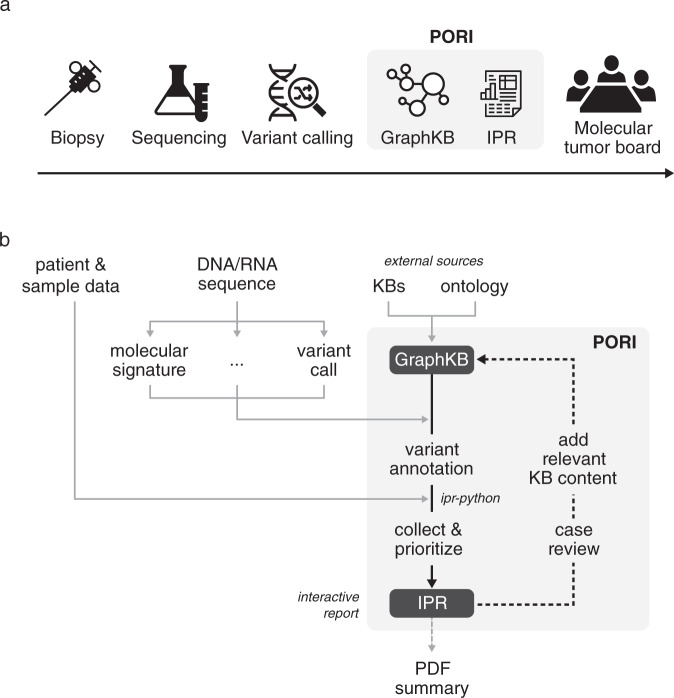
Platform of Oncogenomic Reporting and Interpretation (PORI) overview. PORI Design showing both the placement of PORI within a precision oncology workflow (**a**) and the process of generating a report (**b**). PORI is used for the interpretation and reporting of genomic findings from tumour sequencing. Sequencing Data is taken as input to a number of bioinformatic pipelines and analyses defined by the user. The results of these are loaded by the IPR report python adapter (ipr-python) and annotated with information from GraphKB. After annotation, the results are collated and prioritized based on matches for output into a report using the IPR interactive web platform. This is optionally manually reviewed by the case analyst who may add content to GraphKB as part of their literature review for the case and re-generate the report to include the newly added content. This report is shared with the molecular tumour board (MTB) to inform clinical decisions.

The Platform for Oncogenomic Reporting and Interpretation (PORI) was developed to facilitate the automated analysis of whole-genome and transcriptome sequencing data from human cancer samples to support precision oncology^23^ research initiatives (Fig. 1a). By providing an open-source reporting platform that can be shared and improved by stakeholders, we aim to enable consistency in reporting and reduce redundancy in the development of individual bespoke tools. The PORI platform consists of two main components: a knowledge base (GraphKB) and a reporting tool, Integrated Pipeline Reports (IPR).

**Fig. 2:**
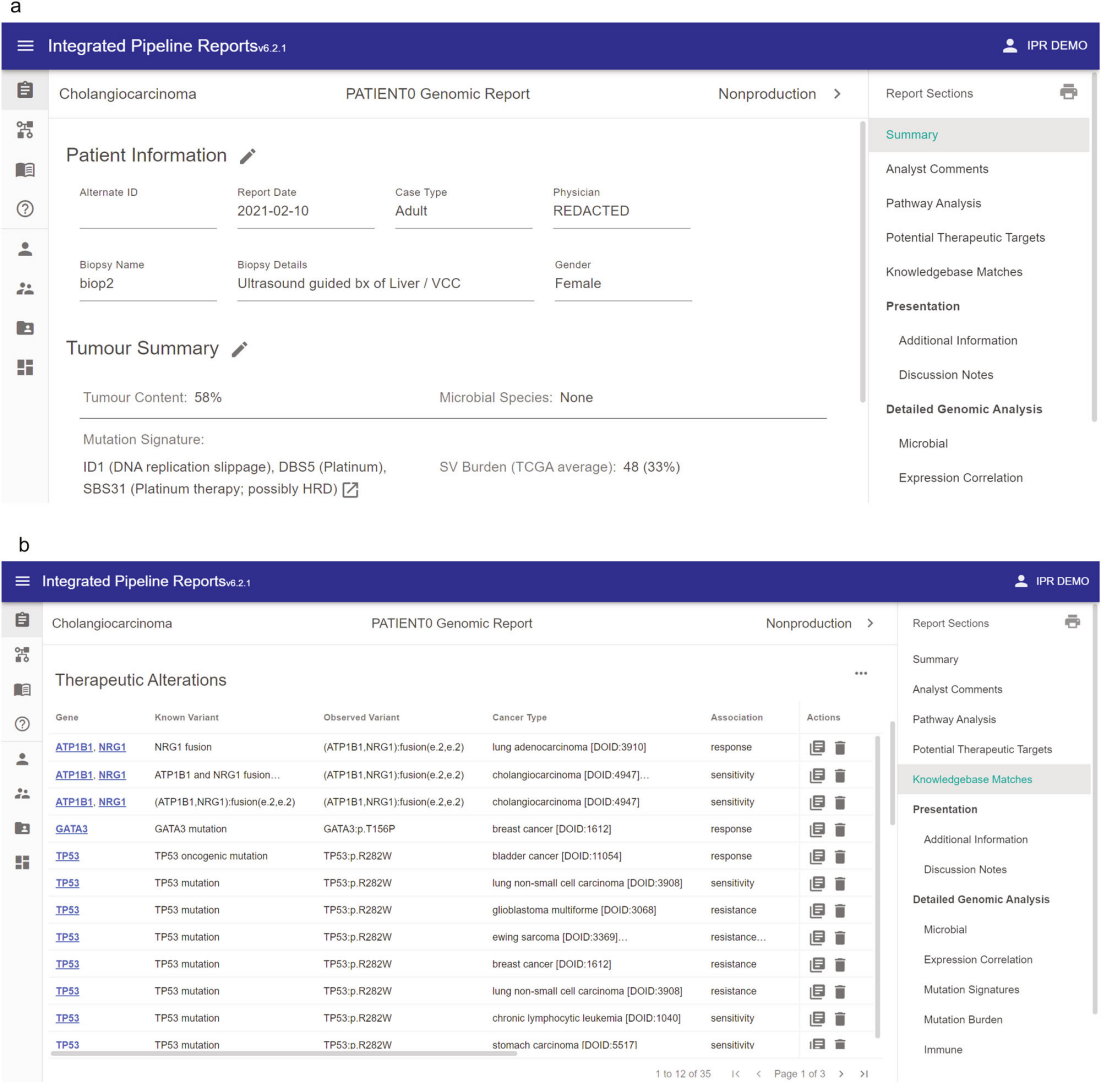
Integrated Pipeline Reports (IPR) web interface. (**a**) The report front page of an example report displaying patient metadata and a summary of tumour characteristics and findings. (**b**) The annotations collected from GraphKB are listed in the knowledge base matches section of the generated report with links from each match back to its corresponding statement in GraphKB.

The knowledge base component, GraphKB, is primarily used to relate variants derived from patient data to known annotations in the literature. The underlying graph structure fundamental to its design enables incorporation of disease, drug, and gene ontologies, biological evidence statements and therapeutic implications from a large number of external databases^3–6,24–29^. There are two ways for the user to interact with GraphKB, via the application programming interface (API) or the web client (Supplementary Fig. 1). As a part of a standard precision oncology workflow, GraphKB annotates patient variants via the same python module used to create a report (Figs. 1b and 2).

The design of GraphKB builds on previous work in aggregating knowledge base content^12^ with several innovations including supporting: mixed manual and automated content; complex variant types; and multiple semi-redundant ontologies. GraphKB is both a standalone knowledge base implementation as well as an aggregate solution or any combination therein. The advantage of this duality is the increased control over the data. Users reap the benefits of consuming and combining multiple external data sources^12^ without sacrificing the ability to manually intervene. This circumvents the need to wait for external updates which can be critical to a quick turnaround time. From the perspective of what is stored, GraphKB goes beyond genomic variants and supports any number of variant types such as expression variants, and molecular signatures.

The reporting component of PORI, IPR, is a web application for the visualization and dissemination of the genomic analysis and corresponding graphics, as well as evidence provided by the integration with GraphKB. It is used to review and communicate data both through the interactive web application as well as the production of portable document format (PDF) summaries (Fig. 1b) suitable for dissemination of research reports to clinical personnel.

GraphKB and IPR are highly integrated. This integration is designed to facilitate the curation of clinically relevant content such as therapeutic biomarkers encountered during literature review of a patient’s variants. Reports are generated against a live version of GraphKB. Content relevant to a given case that was found through literature review and is not already curated in GraphKB can be added during case analysis and the report immediately re-generated. The quick turnaround time (~10 min) and minimal input requirements promote the updating of the knowledge base during case analysis which reduces the workload on the analyst by improving content coverage and consistency between reports. Additionally, inclusion of knowledge base entries into the report motivates the review of existing content; as the analyst reviews the report, they are linked from the report directly to the entries which have been matched in the knowledge base (Fig. 2). This encourages relevant content to be accurate and up to date, as it is reviewed and added with the highest priority.

In order to achieve a comprehensive understanding of a given patient’s disease profile, the integration of diverse types of genomic alterations and complex signatures is required^30^. IPR collects output from many different types of bioinformatic analyses in a single report (Fig. 1b). This provides the user with a central interface to interpret and interact with the data. To maintain flexibility, and recognizing the diversity of existing variant calling pipelines and workflows^22^, the PORI platform is run post-variant calling. In addition to the standard variant calls (SNVs, indels, structural variants, copy variants) PORI supports a number of other analyses including gene expression, mutation signatures, tumour mutation burden, CIBERSORT^31^, MiXCR^32^ and OptiType^33^. A full list of the possible inputs to PORI can be found in the user documentation (https://bcgsc.github.io/pori).

Bioinformatics has a well-known software modality where tools are presented as proof of concept rather than production ready^34^. We have addressed this in PORI with standard techniques such as unit and integration tests using continuous integration and delivery systems. Additionally, PORI has been developed with multiple rounds of user testing. As a part of the Personalized OncoGenomics (POG) program (NCT02155621), PORI has been refined based on feedback from three main user groups: clinicians, clinical trial nurses, and bioinformatic analysts^35^. PORI has been used to generate and review 798 reports by 16 different authors covering 171 different diagnoses (Supplementary Fig. 2).

### GraphKB improves concordance of knowledge base sources

**Fig. 3:**
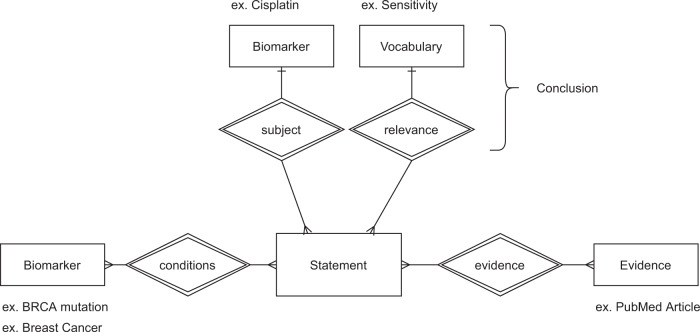
GraphKB Statement Schema. Statements are composed of four main elements: conditions, subject, relevance, and evidence. A statement may be linked to any number of conditions but only one subject and relevance. The conclusion of a statement is considered to be composed only of the relevance and subject.

An expanding number of cancer knowledge bases have become publicly available, providing an opportunity for aggregating and integrating externally curated clinical and biological knowledge into cancer genomic analysis. Due to the variability in the structure and content, integration is necessary to ensure coverage of all relevant annotations^12^. GraphKB is able to support loading content from multiple external knowledge bases as well as adding content directly. Loading tools have been written for several popular knowledge bases which are included in the following knowledge base concordance analysis (Supplementary Table 1): OncoKB^3^; CIViC^4^; COSMIC^6^ (resistance mutations); Cancer Genome Interpreter^5^; and DoCM^36^. Each knowledge base contains both unique and redundant information pertaining to the therapeutic, diagnostic, prognostic and biological relevance of cancer-associated variants. The content of these was compared to determine concordance between knowledge base sources. This was done at the level of conclusions, where conclusions are considered as the relevance (eg. sensitivity or resistance) and subject (ex. Drug or drug class) of a statement (Fig. 3).

Before normalizing, there were 769 unique clinically informative (therapeutic, diagnostic, and prognostic) conclusions. After subject and relevance terms were normalized using ontology relationships, there were 696 unique conclusions, which demonstrates that while the different sources may appear initially to have disparate content, some of that content is in fact shared but done so with alternate representations such as aliases. By normalizing content using the graph model we are able to better quantify the levels of concordance.

**Fig. 4:**
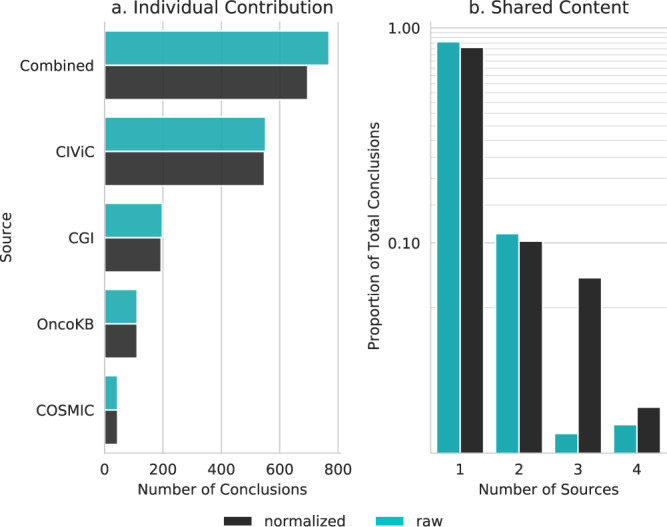
Clinically informative conclusion agreement across knowledge bases. (**a**) The individual contribution of each source is shown as the number of unique conclusions which are given for both raw and normalized counts. The raw values represent the number of conclusions prior to normalization. (**b**) The amount of content which is shared between sources is shown as a fraction of the total number of unique conclusions. Source data are provided as a Source Data file.

The agreement between knowledge base sources increased with normalization of related terms (Fig. 4) from 14% (raw) to 19% (normalized) of conclusions shared in more than 1 source.

### Integrating multiple overlapping ontologies improves ability to incorporate external clinical resources

The ability to leverage existing clinical resources, such as clinical trial registries, represents one of the most enticing use cases of knowledge base content. However, many of these resources do not use ontologies or even controlled vocabulary. In order to import a clinical resource and therefore relate them to patient data, knowledge base controlled vocabulary is matched to the terms used by the clinical resource during import. This process is highly dependent on the controlled vocabulary in the knowledge base covering the terms used by the clinical resource. To demonstrate an application of this, the terms from several widely used disease and drug ontologies (Supplementary Table 2) were compared to disease and drug terms listed in the ClinicalTrials.gov database (https://clinicaltrials.gov), a registry for clinical trials around the world that stores metadata regarding the trial including location, therapy, eligibility criteria, and phase.

**Fig. 5:**
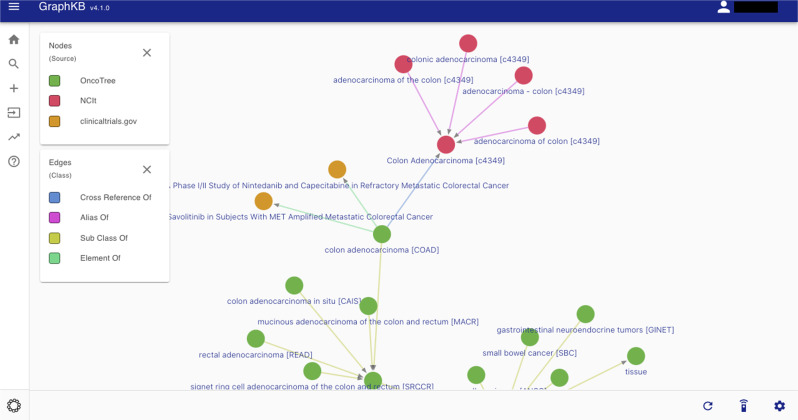
Graph view of content in the GraphKB web application. A subset of links between disease terms related to colorectal adenocarcinoma are shown from NCI thesaurus (NCIt) and OncoTree. For brevity, only a small number of links to clinical trials (Clinicaltrials.gov) are shown.

There are many competing ontologies and standards to choose from. In contrast to other knowledge bases, GraphKB does not enforce a single preferred or standard ontology to be used. While GraphKB does standardize the structure of the entries from external knowledge bases during import, it tries to match the original terminology and specificity. This decreases the number of assumptions that are required while coming at the cost of increased storage size. In order to accomplish this, GraphKB integrates multiple semi-redundant ontologies which are cross-referenced to one another using the links defined by each dataset (Fig. 5). Traditional relational databases are ill-suited to storing this hierarchical data or highly-related data due to the prohibitively high cost in time of joining so many relations. However, graph databases are designed with the connections between the data as a primary focus which allows complex relational queries to be performed efficiently^37^. This ability is leveraged heavily in GraphKB. Ontologies are used as controlled vocabulary, but also to resolve redundant or related terminology through the linking of terms in and between ontologies (Supplementary Fig. 3).

To demonstrate the benefit of including multiple ontologies, we have compared the number of clinical trials terms matched by a single resource to that which we are able to match when resources are combined. Three disease resources were selected: OncoTree (http://oncotree.mskcc.org); Disease Ontology^27^; and NCI Thesaurus (NCIt: https://ncithesaurus.nci.nih.gov). Four drug resources were selected: Food and Drug Administration Substance Registration System (FDA: https://fdasis.nlm.nih.gov/srs); DrugBank^28^; ChEMBL^29^; and NCIt. We define the primary terms of a resource as only the preferred names (as defined by the resource itself) of terms which were given a unique identifier within the resource, excluding aliases, synonyms, or product aliases. Both the primary and full set of terms (indicated hereafter with a+) were used for each resource (Supplementary Table 3). By comparing common names between the full-term sets of each resource, we observed that more than 90% of disease and drug terms were unique to a single resource (Supplementary Fig. 4). This indicates that the total number of terms would be drastically reduced with the use of a single ontology.

**Fig. 6:**
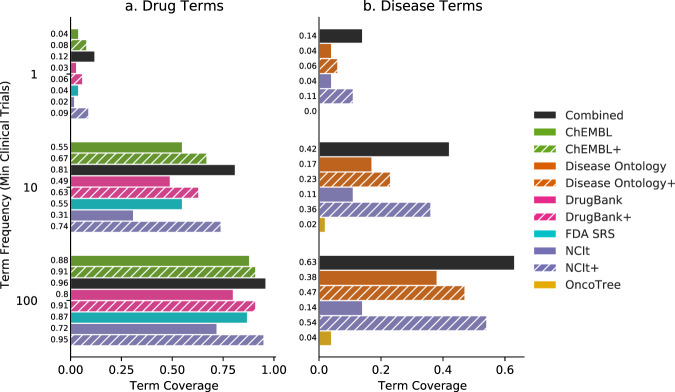
Coverage of clinical trial terminology in popular ontologies. Proportion of matched drug (**a**) and disease (**b**) terms from clinical trials matched by ontology terms across multiple resources. A distinction was made between the primary/preferred terms for a given resource and the set of all terms (indicated with a+), which included synonyms, aliases, and commercial product names. The proportion of total clinical trials terms where an exact match was found in a given ontology is termed coverage. Coverage was calculated for trial terms at 3 frequencies (1+, 10+, or 100+) where the frequency is calculated as the number of clinical trials a given term was used in. Source data are provided as a Source Data file.

Terms were then extracted from the clinical trial records resulting in 116,237 therapy terms and 86,204 disease terms from 345,760 clinical trials (See Methods). Clinical trial terms used at a high frequency in ClinicalTrials.gov (used in >=100 clinical trial records) had higher coverage across ontology resources compared to terms that were less frequently used (Fig. 6). The greatest coverage of clinical trial terms was achieved by combining ontology terms from all resources compared to any single resource (diseases: 0.63 and drugs: 0.96). Among the individual resources, NCIt terms were associated with the greatest coverage of any resource in isolation (diseases: 0.54; drugs: 0.95). However, when only primary terms were considered, ChEMBL (0.88) outperformed the other sources: FDA SRS (0.87); NCIt (0.72); and DrugBank (0.80). Similarly, when only primary terms were considered, the Disease Ontology (0.38) outperformed NCIt (0.14) and OncoTree (0.04). The 9% (7,758 diseases) improvement in disease term coverage of frequently used terms (100+) shows a clear benefit from the inclusion of multiple sources.

### Application of PORI using external data demonstrates the benefit of integration of multiple data types

**Fig. 7:**
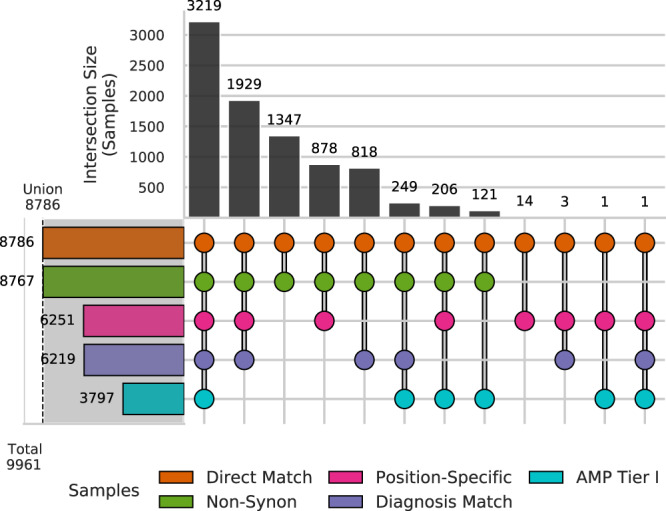
Division of the therapeutic matches to the TCGA samples (*n* = 9,961). The total set of matches is further subdivided by a number of filters. Samples were considered disease matched when the diagnosis of the patient matched the disease listed by the annotation (Diagnosis Match) Samples were considered position matched (Position-Specific) when matches to non-specific gene-level small mutations were excluded. Other filters included: matches with AMP Tier I compatible evidence (AMP Tier I); matches excluding those obtained by second-pass or inferred matching (Direct Match); and finally only non-synonymous, at the protein level, mutations (Non-Synon). The union of all matches is given by the shaded portion and vertical dashed line. Source data are provided as a Source Data file.

To demonstrate the flexibility of PORI both in using external data and supporting multiple data types, we analyzed the TCGA pan cancer atlas cohort^38^. Open-access data files were downloaded from cBioportal.org and analysed using the PORI platform^39,40^. Mutations (mut); copy number variants (cnv); gene expression outliers (exp); and fusions (fus) from all studies were matched to GraphKB and annotated (see Methods). Across all TCGA studies, there were 37,916 unique expression outliers (20,110 increased expression and 17,806 reduced expression); 28,272 unique protein-coding small mutations; 50,124 unique copy variants (25,109 amplifications and 25,015 deep deletions); and 527 unique gene fusions from 9,961 samples. There was a median of 23 unique conclusions per sample (1 sample per report), and a median of 12 conclusions which were therapeutically informative (Supplementary Fig. 5). Of these 9,961 samples (Supplementary Data 1), 8,786 (88.2%) had variants which matched to one or more therapeutic statements and 3,797 (38.1%) were matched to a Tier I AMP evidence level. The full set of therapeutic statements includes experimental, pre-clinical, and investigational biomarkers whereas AMP Tier I filtered statements are limited to FDA-approved treatments. When accounting for disease type, 6,219 (62.4%) samples had therapeutic statements of which 3,469 (34.8%) matched to a tier I AMP evidence level (Fig. 7). These cases were further analyzed as these represent potential therapeutic interventions or recommendations.

**Fig. 8:**
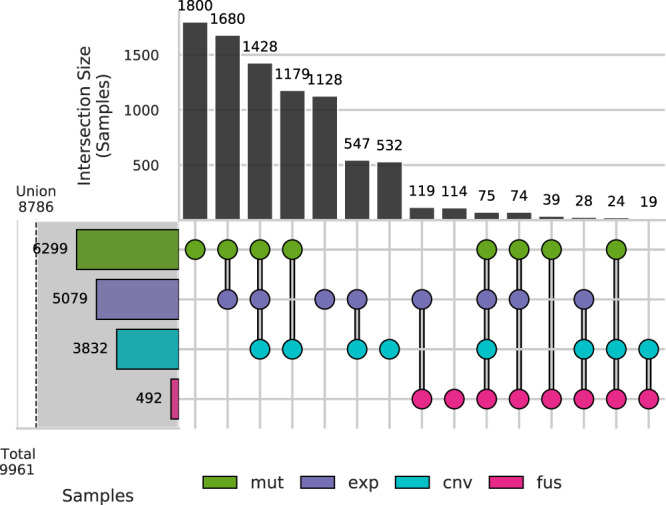
Proportion of samples (*n* = 9,961) with therapeutic matches derived from each combination of variant types. Upset plot of the number of samples with therapeutic matches from annotation of a given variant type. Sample variants are divided into four types: copy number variants (cnv); single nucleotide variants and indels (mut); gene fusions (fus); and gene expression (exp) variants. The left-hand bar plots are the total number of samples which have 1 or more therapeutic conclusions matched to the listed variant type. The union of all matches is given by the shaded portion and vertical dashed line. The upper bar plots show the number of samples in each of the intersection groups. These groups are mutually exclusive. Source data are provided as a Source Data file.

A large proportion of samples had therapeutic matches derived from a single variant type (small mutations: 18.1%; RNA expression: 11.3%; copy number: 5.34%), which demonstrates the importance of the inclusion of multiple variant types. If we only included a single variant type for GraphKB matching and reporting, then the number of samples where no therapeutic matches were found would increase by a minimum of 2,487 samples (25.0%) depending on which variant type was selected (Fig. 8).

As expected, since small mutations have the greatest coverage across all public knowledge base sources used in this analysis, we observed the greatest contribution to therapeutic matches from statements associated with this variant type (Fig. 8). However, expression-based variants, as defined by a combination of z-score and percentile thresholds (z score −2/+2 and percentile 2.5/97.5, respectively), led to a greater proportion of patients with a therapeutic match compared to both fusion and copy number variants. 1,128 samples (11.3%) had therapeutic matches derived solely from expression data. This highlights an opportunity for greater focus on expression variants and their clinical or biological significance.

### PORI identifies therapeutically relevant alterations in a cholangiocarcinoma patient

To demonstrate the use of PORI for clinically relevant interpretation of individual patient data, we analysed a case of cholangiocarcinoma, which was previously described as harbouring a fusion involving the oncogene *NRG1*^41^. The patient, a 38-year old woman diagnosed with intrahepatic cholangiocarcinoma, had received chemotherapy with gemcitabine and cisplatin and undergone surgery, without disease control. A metastatic tumour sample was obtained from the liver and analysed using whole-genome and transcriptome sequencing, revealing small mutations, copy number changes, structural variants, and gene expression alterations. An *ATP1B1-NRG1* gene fusion was identified which led to the rationalization for treatment with the ErbB family tyrosine kinase inhibitor afatinib, with dramatic subsequent clinical response^41^. The data from this analysis, which was processed with PORI, including matching to graphKB and display in IPR, has been made available at https://bcgsc.github.io/pori/demo (IPR, PATIENT0 biop2).

PORI clearly identified the key targetable alteration, the *NRG1* fusion, on the summary page (Supplementary Fig. 6) based on matching to therapeutically relevant statements in GraphKB, along with the display of a figure describing the structural variant^42^ (Supplementary Fig. 7). In addition, mutations in tumour suppressors *TP53* and *CDKN2A* are highlighted, along with amplification of the oncogenes *NTRK1* and *MCL1*. A number of genes have notably increased expression, including *NRG1*, consistent with the oncogenic effect of the gene fusion, and expression information can be viewed, sorted and filtered within IPR (Supplementary Fig. 8). Details of the GraphKB associations provide information on drug sensitivity, resistance, and eligibility for clinical trials, as well as tumour type information and links to the source data in GraphKB. This provides critical support for an informed decision about therapy options, including in this case the potential for sensitivity to afatinib, which was procured and resulted in a clinical response for this patient. In addition to specific gene associations, mutation signatures analysis^43^ (Supplementary Fig. 9) reveals that this sample harbours evidence of exposure to platinum therapy (SBS31 and DBS5) consistent with the treatment history of the patient. While reports may be generated with a minimal input of somatic mutations, PORI provides flexibility for the addition of other data types when available from user-defined analysis pipelines, including expression correlation (Supplementary Fig. 10), immune environment, and mutation burden. The interactive nature of IPR allows the user to quickly view the genomic events associated with the strongest evidence of clinical relevance, and to also access the level of detail that is most pertinent, supporting informed treatment decision-making for precision oncology.

## Discussion

The rapid development of genomic technologies and bioinformatic research represents a significant challenge for precision oncology^30^. Platforms and pipelines must be able to readily incorporate new and varied content. PORI addresses this with modular reports where sections corresponding to particular specialized analyses can be added or removed as available. Previous reporting solutions have required users to input raw data and use the bioinformatic analysis pipeline integrated into the tool itself^17^. This is a barrier to use for many institutions which have already developed their own mature bioinformatic pipelines. It also limits the ability of the user to modify the pipeline as new tools are developed and further data types are added. PORI overcomes this by requiring inputs post-variant calling. Automation often comes at the cost of fine-grained control over the product. While fully automated solutions have shown promise for very common cancer types, their success with less common cancers has demonstrated there is still a strong need for human expert intervention^44^. PORI balances this by generating a fully automated report which can be manually altered and supplemented as needed.

The importance of an open-source platform is three-fold. Firstly, due to the flexible design of PORI, users will be able to contribute content as needed both in the form of additional loaders for GraphKB and additional sections for new analysis types in IPR. Community involvement will help ensure the reporting platform continues to support relevant inputs that reflect the needs of the community. Secondly, the provision of a transparent option for reporting genomic data will provide an opportunity to standardize and improve reporting across multiple centres which will facilitate simpler comparisons. This is particularly critical as it has been shown that commercial platforms provide diverse results that are less amenable to scrutiny^15^. Finally, this will provide access to institutions and centres which might find the commercial alternatives cost-prohibitive.

Significant progress has been made in the area of somatic variant interpretation for application in precision medicine. The ability to apply reproducible, evidence-based definitions of clinically actionable variants is a major area of research in the field. One method that has been employed in the assignment of evidence levels, based on the current landscape of published literature for a given variant, resulting in the development of several different evidence tiering schemes. A landmark joint publication from ASCO, AMP and CAP in 2017 laid a framework for somatic variant interpretation and evidence tiering, which has been used in further initiatives, including the Variant Interpretation for Cancer Consortium, aimed at harmonizing evidence levels from multiple sources^12,45^. GraphKB is well-suited to accommodate multiple evidence schemes, enabling the generation of relationships between evidence tiers from individual resources. Integration of GraphKB statements and corresponding evidence tiers enables the end-user to assess the strength of evidence for a given variant and knowledge base statement. For example, across the TCGA cohort, we noted a relatively high proportion of samples had at least one therapeutic statement matched by genomic, or transcriptomic data (88.2%) representing experimental, off-label, and pre-clinical treatment options. Recent work in a precision oncology clinical trial by the German Cancer Consortium has found a similar rate of experimental biomarkers in their patient population^46^. While specific clinical trials are able to treat patients with this lower tier evidence^46,47^, in practice, only a subset of matches corresponding to the disease-matched AMP tier I evidence level statements (34.8% in this data set) are likely to be able to be clinically acted upon. By incorporating evidence levels from multiple sources and the ability to map between them, a greater number of therapeutic statements can be readily assessed through the integration of GraphKB with IPR in the PORI platform.

While integrating several resources improves coverage of the knowledge space, the observation that the majority of evidence statements are only represented in a single resource suggests existing resources only capture a fraction of current knowledge. This problem is exacerbated by an increasing number of knowledge bases with restrictive licensing that does not permit re-use or sharing. Public open data initiatives, like CIViC^4^, are therefore increasingly important both in their ability to contribute accessible data but also their advantage in crowd-sourcing curation to achieve a larger number of annotators. While we encourage PORI users to contribute to these public resources, we recognize that it is not always immediately possible. Knowledge may be pre-publish or not yet supported by the public knowledge bases (ex. complex variants). Future work on PORI will include collaborating with external open-data community members to automate the export of entries annotated privately in instances GraphKB when users are ready to contribute them to public portals.

Although PORI represents an important first step in creating an open-source standard tool for reporting in precision oncology, there are still many avenues for future development. Currently, the platform focuses on creating research reports and future iterations could include clinically accreditable formats of the report. Work is currently underway to create germline and pharmacogenomic report variants. Finally, perhaps the most exciting area for future work is in the application of data captured from user actions during the analysis process to iteratively improve and further automate future analysis. As more knowledge is curated, each patient will match more annotations and reports will become increasingly verbose. There will be an increased need to succinctly and efficiently summarize the implications for a given patient. This will involve condensation of statements not only from different resources but also across multiple matched variants. Future work on IPR may leverage the interactive nature of IPR to explore and evaluate partially automated summarization via machine learning algorithms. Facilitating the complex analysis associated with precision oncology in cancer will not only have direct benefit to the patients analyzed but also the process as a whole through improved communication and transparency.

## Methods

### GraphKB Transformation of sources for Knowledge Base Comparison

#### Import into GraphKB

Knowledge Base Data is imported into GraphKB via automated scripts which can be found in our loader repository (Supplementary Table 4). While there is some logic specific to each source, in general the logic is that ontology terms are imported from multiple sources. Cross reference links are imported where defined and the ontology that defines the linkage is set to the source of the link. Knowledge bases are imported after ontologies as many of them require the ontologies as dependencies. For terms referenced in a knowledge base from a particular ontology the statement is linked to the specified ontology. If an ontology was not given then the term is matched by exact name match or an error is reported (Supplementary Table 1).

To ensure this process is traceable and repeatable each ontology field is stored with four main inputs: source, sourceId, name, and sourceIdVersion. The source is the ontology it was imported from (ex. HGNC). The sourceId is the Id defined by the source, this should be unique within the source (ex. 6407). The name is the human readable name of the term (ex. KRAS). and finally the sourceIdVersion is the version number of that Id. This field is optional. In some cases this may be the same as the version number of the entire resource but in many the IDs themselves are versioned independently (ex. ensembl transcript versions).

### Processing of resources for ontology term name comparisons

The set of unique drug (or disease) names defined by each resource as well as any synonyms or product names was taken. These have been transformed to lowercase and trimmed.

#### ClinicalTrials.gov clinical trials

The full XML records for all trials (346,614) were downloaded on 2020-07-23 from ClinicalTrials.gov [https://clinicaltrials.gov/AllPublicXML.zip]. From these, conditions and interventions were parsed into a list of terms and the frequency amongst trials of these terms. Interventions of the following types were considered drug terms: Drug, Radiation, Combination Product, or Dietary Supplement. Normalization of all terms was limited to stripping trailing and leading whitespace and lowercasing. This resulted in 116,237 therapy terms and 86,204 disease terms from 345,760 clinical trials.

#### NCIt

The plain text download version of the NCIt thesaurus was downloaded from NCIt [https://evs.nci.nih.gov/ftp1/NCI_Thesaurus/archive/2020/20.06e_Release/Thesaurus.FLAT.zip]. Terms were classified as disease or therapy based on their semantic type (Supplementary Table 3). Terms with the following semantic types were considered therapeutic terms (87,427 total; 5,017 primary): Antibiotic; Biologically Active Substance; Biomedical or Dental Material; Chemical Viewed Functionally; Chemical Viewed Structurally; Chemical; Clinical Drug; Drug Delivery Device; Element, Ion, or Isotope; Food; ‘Hazardous or Poisonous Substance; Hormone; Immunologic Factor; Indicator, Reagent, or Diagnostic Aid; Inorganic Chemical; Medical Device; Organic Chemical; Pharmacologic Substance; Plant; Steroid; Substance; Therapeutic or Preventive Procedure; and, Vitamin. Terms with the following semantic types were considered disease terms (57,276 total; 6,526 primary): Anatomical Abnormality; Congenital Abnormality; Disease or Syndrome; Experimental Model of Disease; Mental or Behavioral Dysfunction; Neoplastic Process; Sign or Symptom. Both the names and synonyms of the terms were considered.

#### DrugBank

DrugBank^28^ (v5.1.7) was downloaded in its XML format [https://go.drugbank.com/releases/5-1-8/downloads/all-full-database]. Names were extracted from the records based on the name, synonyms, and products tags. This resulted in 131,412 unique terms (Supplementary Table 3).

#### FDA SRS

The UNII identifiers (version 27Mar2020) were downloaded from the FDA substance registration system [https://fdasis.nlm.nih.gov/srs/download/srs/UNIIs_20200327.zip]. The PT field was used as the name field. This resulted in 109,334 primary terms (all terms have unique identifiers and therefore are considered non-alias primary terms) (Supplementary Table 3).

#### Disease ontology

The disease ontology^27^ (v2020-06-18) was downloaded as a JSON [https://github.com/DiseaseOntology/HumanDiseaseOntology/blob/v2020-06-18/src/ontology/releases/2020-06-18/doid.json]. Terms were extracted from the lbl and synonyms attributes. This resulted in 19,064 primary terms out of 38,489 total terms (Supplementary Table 3).

#### ChEMBL

The postgres dump of the ChEMBL^29^ (version 27) database was downloaded [https://chembl.gitbook.io/chembl-interface-documentation/downloads; 10.6019/CHEMBL.database.27] and a plain text version of the drug names was created from the molecule_dictionary and molecule_synonym tables. Where the preferred name field of the first table was used as the primary set of terms and names from the synonyms table were included in the full set (Supplementary Table 3). This resulted in 35,219 primary terms out of 123,287 total terms.

### Processing of TCGA data

All TCGA pan-cancer ATLAS data was downloaded from cBioportal.org^39^. This consisted of the all *_tcga_pan_can_atlas_2018 studies: BLCA, BRCA, CESC, CHOL, COADREAD, DLBC, ESCA, GBM, HNSC, KICH, KIRC, KIRP, LAML, LGG, LIHC, LUAD, LUSC, MESO, OV, PAAD, PCPG, PRAD, SARC, SKCM, TGCT, THCA, THYM, UCEC, UCS, and UVM. Data was processed from all studies (Supplementary Data 1) with the exception of LUAD which was found to be incomplete and therefore not processed further.

Variants were compiled for each sample (includes some repeat samples from the same patient) from the expression (data_RNA_Seq_v2_mRNA_median_all_sample_Zscores.txt; TCGA only); copy number (data_CNA.txt); small mutations (data_mutations_extended.txt); and fusions (data_fusions.txt) files. Copy variants were classified as deep deletion with a value of −2 or lower and amplification with a value of 2 or greater. The distribution of copy number values amongst all patients was plotted as a sanity check that these values would result in outliers and not represent a large percentage of the calls (Supplementary Fig. 11). The expression z-scores are pre-calculated in the cBioportal data. A threshold combination of z-score (−2, +2) and percentile (2.5, 97.5) was used in evaluating expression variants to determine outliers (See related demo for calculating RNA metrics: https://bcgsc.github.io/pori/ipr/scripting/RNA_Expression_Metrics). Small mutations and fusion matching was limited to genes and mutations with protein changes (for small mutations). For simplicity, matching did not include intergenic mutations.

The number of variants called per sample (*n* = 9,961) was compared across all studies for each variant type (Supplementary Fig. 12) which had median values of: 402 expression variants; 0 gene fusions; 84 copy variants; and two small mutations. A one-way ANOVA was performed to determine significance (F-statistic: 4613.24, *p* value: 0.0) followed by a TukeyHSD test to investigate pairs. The null hypothesis was rejected for all combination pairs within a data set (FWER = 0.05, adjusted *p* value of 0.001) with the exception of fusions and small mutations which had a *p* value of 0.9.

Each variant was then matched to GraphKB using the GraphKB python adapter (Supplementary Table 4). From these statement matches, the number of matches by variant type per each conclusion was determined. As second-pass matching (https://bcgsc.github.io/pori/faq/#what-is-second-pass-matching) may include multiple variant types, these matches were excluded from the variant type analysis. The conclusion of a statement is considered to be the combination of its relevance and subject fields (Fig. 3).

These matches were then refined by five sets of filters to further characterize their therapeutic applicability: Association for Molecular Pathology (AMP) evidence level Tiering (AMP Tier I), disease match status (Diagnosis Match), positional specificity (Position-Specific), direct matching only (Direct Match), and non-synonymous protein change (Non-Synon) (Fig. 7)

#### AMP tier I filter

To create the AMP Tier I filter, evidence levels were mapped to AMP Tiers^12^. Within each knowledge base analyzed the following evidence levels were considered AMP Tier I: CIViC (A); CGI (FDA guidelines, NCCN guidelines, CPIC guidelines, NCCN/CAP guidelines, and European Leukemianet guidelines); and OncoKB (1, R1, 2A). For knowledge bases without an evidence scheme (ex. COSMIC) no statements were considered to be AMP Tier I. Following this matches were restricted to only statements which contained an AMP Tier I compatible evidence level.

#### Diagnosis match filter

Diseases were considered matched (Diagnosis Match) when the term given in the knowledge base statement matched terms collected on expanding the diagnosis terms (Supplementary Fig. 3) in the sample metadata. The OncoTree code, detailed cancer type, and cancer type were expanded to collect the final set of diagnosis disease terms.

#### Position specific filter

To facilitate comparison with other related analyses, the position-specific filter was created. This filter excludes all statements which were matched based on a non-specific small mutation. This would exclude any statements with small mutation variants that did not give a position by HGVS. For example, a statement with the variant oncogenic mutations in KRAS would be excluded.

#### Direct match filter

GraphKB enables matching via second-pass or indirect matching (https://bcgsc.github.io/pori/faq/#what-is-second-pass-matching) where an initially matched variant may match a statement which characterizes that variant functionally (ex. oncogenic mutations) and using the initially matched statement a second statement which builds on the functional characterization (ex. oncogenic mutations in KRAS are associated with resistance to EGFR inhibitors) is matched. The direct filter excludes all second-pass or indirect matches.

### Sequencing and analysis of the cholangiocarcinoma case

#### Tumour sampling, library construction and sequencing

An ultrasound guided biopsy of the liver metastasis was collected from the patient, a 38 year-old woman^41^. It was then embedded in an optimal cutting temperature compound, and sectioned. A peripheral blood sample was taken representing a normal cell sample from the patient. DNA and RNA were purified using the AllPrep DNA/RNA Mini Kit (Qiagen). PCR-free DNA libraries were constructed by shearing by sonication (Covaris), end-repair and size selection using AMPure XP beads targeting a 300–400 bp fraction, 3ʹ A-tailing, ligation using full length TruSeq adapters, and purification using AMPure XP beads. PolyA+ RNA was purified using the MultiMACS mRNA isolation kit (Miltenyi Biotec, Germany), first-strand cDNA synthesized using the Maxima H Minus First Strand cDNA Synthesis kit (Thermo-Fisher, USA) with random hexamer primers, and second strand cDNA synthesized following the Superscript cDNA Synthesis protocol. The RNA library was constructed by fragmentation of cDNA by sonication (Covaris), purification using Ampure XP SPRI beads, end-repair, phosphorylation, 3ʹ A-tailing, ligation using Illumina adapters, and purification using Ampure XP SPRI beads, followed by PCR amplification using Illumina’s PE primer set. Genome and transcriptome sequencing was performed on HiSeq2500 instruments (Illumina, San Diego, California) using paired-end reads. The tumour DNA was sequenced with 150 bp reads to 79X average coverage, the normal DNA with 125 bp reads to 46X average coverage, and the RNA sequenced to 248 million reads trimmed to 75 bp.

#### Somatic alterations

DNA reads were aligned to human reference hg19 using the BWA tool (v0.5.7)^46^. Tumour-specific copy variants were identified with CNAseq (v0.0.6)^47^ and regions of LOH using APOLLOH (v0.1.1)^48^. Structural variants were identified using ABySS (v1.3.4)^49^, Trans-ABYSS (v1.4.10)^50^, DELLY (v0.6.1)^51^, Manta (v1.0.0)^52^, and DeFUSE (v0.6.1)^53^. These calls were then combined and validated using MAVIS (v2.1.1)^42^. Somatic single nucleotide mutations were identified using Strelka (v1.0.6)^54^ and MutationSeq (v1.0.2)^55^. Somatic small insertions and deletions were identified using Strelka and Trans-ABYSS. Variants were annotated using Ensembl gene models (v69)^56^. Mutation signatures were computed using Strelka somatic SNVs categorized into 96 mutation classes^57^, subjected to a non-negative least squares deconvolution based on COSMIC mutation signatures (https://cancer.sanger.ac.uk/cosmic/signatures_v2)^43^. HLA types were determined using OptiType^33^.

#### Gene expression

RNA-Seq reads were aligned using Jaguar (v2.0.3)^58^, from which expression levels in reads per kilobase per million mapped reads (RPKM) were computed. To determine genes with outlier expression for reporting, comparison was made to tumours of The Cancer Genome Atlas (TCGA, https://tcga-data.nci.nih.gov/tcga), normal samples from Illumina Human Body Map 2.0, and the Genotype-Tissue Expression (GTEx) Project (https://www.gtexportal.org), considering percentile, number of interquartile ranges (kIQR) from the median value, and fold-change. Similar tumour types were identified using spearman correlation values to TCGA datasets based on a set of 1,744 genes, and also the machine-learning based classified SCOPE^59^.

### Reporting summary

Further information on research design is available in the Nature Research Reporting Summary linked to this article.

## Supplementary information

Supplementary Information

Supplementary Data 1

Reporting Summary

## Data Availability

The Disease Ontology data used in this study are available from the github repository (v2020-06-18) [https://github.com/DiseaseOntology/HumanDiseaseOntology/blob/v2020-06-18/src/ontology/releases/2020-06-18/doid.json]. The FDA SRS data used in this study are available from the FDA downloads page (March 27 2020 release) [https://fdasis.nlm.nih.gov/srs/download/srs/UNIIs_20200327.zip]. The DrugBank data used in this study are available from the DrugBank releases page (v5.1.8) [https://go.drugbank.com/releases/5-1-8/downloads/all-full-database]. The NCIt data used in this study are available from the NCIt ftp downloads page (20.06e) [https://evs.nci.nih.gov/ftp1/NCI_Thesaurus/archive/2020/20.06e_Release/Thesaurus.FLAT.zip]. The ChEMBL data used in this study are available from the ChEMBL FTP downloads page [10.6019/CHEMBL.database.27]. The OncoTree data used in this study are available from the OncoTree API (oncotree_2020_04_01) [http://oncotree.mskcc.org/api/tumorTypes?version=oncotree_2020_04_01]. The clinical trials data used in this study are available from ClinicalTrials.gov [https://clinicaltrials.gov/AllPublicXML.zip]. The CIViC data used in this study is available from the CIViC API [https://civicdb.org/api]. The OncoKB data are available under restricted access due to licensing requirements, access can be obtained by registering for a license of the OncoKB data [https://www.oncokb.org]. The CGI data used in this study is available from the CGI webpage [https://www.cancergenomeinterpreter.org/data/cgi_biomarkers_latest.zip]. The COSMIC data used in this study is available from the file downloads page of the COSMIC website (v92) [https://cancer.sanger.ac.uk/cosmic/file_download/GRCh38/cosmic/v92/CosmicResistanceMutations.tsv.gz]. The DoCM data used in this study is available from the DoCM API [http://docm.info/api/v1/variants]. TCGA PanCancer Atlas Studies data was accessed and downloaded from cBioportal.org [http://www.cbioportal.org/datasets]. A full list of sample accession numbers is provided in Supplementary Data 1. The Genomic and transcriptomic datasets for the cholangiocarcinoma case study have been previously deposited and are available in the European Genome-phenome Archive under accession number EGAD00001002623. The report for this data is available via the PORI demo (0bdec40b-04d7-4264-aa3f-7ddb4cbeebf5) [https://bcgsc.github.io/pori/demo]. The GTEx datasets used for the cholangiocarcinoma case analyses described in this manuscript were obtained from dbGaP through accession number phs000424.v6.p1 and TCGA data for this case were derived from RNA-Seq gene expression data now available through the Genomic Data Commons Data Portal (https://portal.gdc.cancer.gov/), project names starting with “TCGA-“.The Illumina human body map (2.0) data used for this case is available from the Gene Expression Omnibus under accession number GSE30611. Source data are provided with this paper.

## Source data

Source Data

## Code availability

The user manual of PORI can be found at https://bcgsc.github.io/pori. Implementation and peer review details of code for PORI can be found in the related repositories^60–70^ (Supplementary Table 4) which are summarized in the user documentation for convenience at https://bcgsc.github.io/pori/repos. The central repository [https://github.com/bcgsc/pori] is the access point for those wishing to install their own instance of PORI or update the user documentation. Code for the various components of PORI is organized across several repositories. GraphKB consists of an API [https://github.com/bcgsc/pori_graphkb_api], a web client [https://github.com/bcgsc/pori_graphkb_client], data loaders [https://github.com/bcgsc/pori_graphkb_loader], and shared modules between them [https://github.com/bcgsc/pori_graphkb_parser, https://github.com/bcgsc/pori_graphkb_schema]. IPR consists of an API [https://github.com/bcgsc/pori_ipr_api] and a web client [https://github.com/bcgsc/pori_ipr_client]. Users with access to an existing PORI instance, aiming to incorporate PORI into their own python scripts, can find the code for the python adapters in the following repositories: GraphKB [https://github.com/bcgsc/pori_graphkb_python], and IPR [https://github.com/bcgsc/pori_ipr_python], cBioportal reports [https://github.com/bcgsc/pori_cbioportal].

## References

[1] Good BM, Ainscough BJ, McMichael JF, Su AI, Griffith OL (2014). Organizing knowledge to enable personalization of medicine in cancer. Genome Biol..

[2] Mardis ER (2010). The 1,000 genome, the 100,000 analysis?. Genome Med.

[3] Chakravarty, D. et al. OncoKB: A Precision Oncology Knowledge Base. *JCO Precis Oncol* **2017**, PO.17.00011 (2017). 10.1200/PO.17.00011 PMC5586540 28890946

[4] Griffith M (2017). CIViC is a community knowledgebase for expert crowdsourcing the clinical interpretation of variants in cancer. Nat. Genet..

[5] Tamborero D (2018). Cancer Genome Interpreter annotates the biological and clinical relevance of tumor alterations. Genome Med.

[6] Tate JG (2019). COSMIC: the catalogue of somatic mutations in cancer. Nucleic Acids Res.

[7] Patterson SE (2016). The clinical trial landscape in oncology and connectivity of somatic mutational profiles to targeted therapies. Hum. Genomics.

[8] Huang L (2017). The cancer precision medicine knowledge base for structured clinical-grade mutations and interpretations. J. Am. Med. Inform. Assoc..

[9] Taylor AD, Micheel CM, Anderson IA, Levy MA, Lovly CM (2016). The path(way) less traveled: a pathway-oriented approach to providing information about precision cancer medicine on my cancer genome. Transl. Oncol..

[10] Dumbrava, E. I. & Meric-Bernstam, F. Personalized cancer therapy-leveraging a knowledge base for clinical decision-making. *Cold Spring Harb Mol Case Stud* **4**, a001578 (2018). 10.1101/mcs.a001578 PMC5880252 29212833

[11] Damodaran S (2015). Cancer driver log (CanDL): catalog of potentially actionable cancer mutations. J. Mol. Diagn..

[12] Wagner AH (2020). A harmonized meta-knowledgebase of clinical interpretations of somatic genomic variants in cancer. Nat. Genet..

[13] Goldman MJ (2020). Visualizing and interpreting cancer genomics data via the Xena platform. Nat. Biotechnol..

[14] Zhou X (2021). Exploration of coding and non-coding variants in cancer using GenomePaint. Cancer Cell.

[15] Perakis, S. O. et al. Comparison of three commercial decision support platforms for matching of next-generation sequencing results with therapies in patients with cancer. *ESMO Open* **5**, e000872 (2020). 10.1136/esmoopen-2020-000872 PMC7513637 32967919

[16] Katsoulakis, E., Duffy, J. E., Hintze, B., Spector, N. L. & Kelley, M. J. Comparison of annotation services for next-generation sequencing in a large-scale precision oncology program. *JCO Precis Oncol* **4**, PO.19.00118 (2020). 10.1200/PO.19.00118 PMC7446349 32923873

[17] Meißner T, Fisch KM, Gioia L, Su AI (2015). OncoRep: an n-of-1 reporting tool to support genome-guided treatment for breast cancer patients using RNA-sequencing. *BMC Med*. Genomics.

[18] Nakken S (2018). Personal Cancer Genome Reporter: variant interpretation report for precision oncology. Bioinformatics.

[19] Gray SW (2018). Interactive or static reports to guide clinical interpretation of cancer genomics. J. Am. Med. Inform. Assoc..

[20] Kaplan, B. Seeing through health information technology: the need for transparency in software, algorithms, data privacy, and regulation*. *J Law Biosci* **7**, lsaa062 (2020). 10.1093/jlb/lsaa062 PMC8240841 34350004

[21] Quackenbush J (2003). Open-source software accelerates bioinformatics. Genome Biol..

[22] Corbett RD (2020). A distributed whole genome sequencing benchmark study. Front. Genet..

[23] Laskin J (2015). Lessons learned from the application of whole-genome analysis to the treatment of patients with advanced cancers. Cold Spring Harb. Mol. Case Stud..

[24] Braschi B (2019). Genenames.org: the HGNC and VGNC resources in 2019. Nucleic Acids Res.

[25] Yates AD (2020). Ensembl 2020. Nucleic Acids Res..

[26] O’Leary NA (2016). Reference sequence (RefSeq) database at NCBI: current status, taxonomic expansion, and functional annotation. Nucleic Acids Res.

[27] Schriml LM (2019). Human disease ontology 2018 update: classification, content and workflow expansion. Nucleic Acids Res.

[28] Wishart DS (2018). DrugBank 5.0: a major update to the DrugBank database for 2018. Nucleic Acids Res.

[29] Gaulton A (2017). The ChEMBL database in 2017. Nucleic Acids Res.

[30] Zhang H, Klareskog L, Matussek A, Pfister SM, Benson M (2019). Translating genomic medicine to the clinic: challenges and opportunities. Genome Med.

[31] Newman AM (2015). Robust enumeration of cell subsets from tissue expression profiles. Nat. Methods.

[32] Bolotin DA (2015). MiXCR: software for comprehensive adaptive immunity profiling. Nat. Methods.

[33] Szolek A (2014). OptiType: precision HLA typing from next-generation sequencing data. Bioinformatics.

[34] Mangul S (2019). Challenges and recommendations to improve the installability and archival stability of omics computational tools. PLoS Biol..

[35] Pleasance E (2020). Pan-cancer analysis of advanced patient tumors reveals interactions between therapy and genomic landscapes. Nat. Cancer.

[36] Ainscough BJ (2016). DoCM: a database of curated mutations in cancer. Nat. Methods.

[37] Nayak A (2013). Type of NOSQL databases and its comparison with relational databases. Int J of App Information Syst.

[38] Hoadley KA (2018). Cell-of-origin patterns dominate the molecular classification of 10,000 tumors from 33 types of cancer. Cell.

[39] Gao J (2013). Integrative analysis of complex cancer genomics and clinical profiles using the cBioPortal. Sci. Signal..

[40] Cerami E (2012). The cBio cancer genomics portal: an open platform for exploring multidimensional cancer genomics data. Cancer Disco..

[41] Jones MR (2017). Successful targeting of the NRG1 pathway indicates novel treatment strategy for metastatic cancer. Ann. Oncol..

[42] Reisle C (2019). MAVIS: merging, annotation, validation, and illustration of structural variants. Bioinformatics.

[43] Alexandrov LB (2020). The repertoire of mutational signatures in human cancer. Nature.

[44] Zhou N (2019). Concordance study between ibm watson for oncology and clinical practice for patients with cancer in China. Oncologist.

[45] Li MM (2017). Standards and guidelines for the interpretation and reporting of sequence variants in cancer: a joint consensus recommendation of the Association for Molecular Pathology, American Society of Clinical Oncology, and College of American Pathologists. J. Mol. Diagn..

[46] Li H, Durbin R (2009). Fast and accurate short read alignment with Burrows-Wheeler transform. Bioinformatics.

[47] Jones SJ (2010). Evolution of an adenocarcinoma in response to selection by targeted kinase inhibitors. Genome Biol..

[48] Ha G (2012). Integrative analysis of genome-wide loss of heterozygosity and monoallelic expression at nucleotide resolution reveals disrupted pathways in triple-negative breast cancer. Genome Res..

[49] Robertson G (2010). De novo assembly and analysis of RNA-seq data. Nat. Methods.

[50] Birol I (2009). De novo transcriptome assembly with ABySS. Bioinformatics.

[51] Rausch T (2012). DELLY: structural variant discovery by integrated paired-end and split-read analysis. Bioinformatics.

[52] Chen X (2016). Manta: rapid detection of structural variants and indels for germline and cancer sequencing applications. Bioinformatics.

[53] McPherson A (2011). deFuse: an algorithm for gene fusion discovery in tumor RNA-Seq data. PLoS Comput. Biol..

[54] Saunders CT (2012). Strelka: accurate somatic small-variant calling from sequenced tumor–normal sample pairs. Bioinformatics.

[55] Ding J (2011). Feature-based classifiers for somatic mutation detection in tumour–normal paired sequencing data. Bioinformatics.

[56] Flicek P (2014). Ensembl 2014. Nucleic Acids Res.

[57] Alexandrov LB (2013). Signatures of mutational processes in human cancer. Nature.

[58] Butterfield YS (2014). JAGuaR: junction alignments to genome for RNA-seq reads. PLoS One.

[59] Grewal JK (2019). Application of a neural network whole transcriptome–based pan-cancer method for diagnosis of primary and metastatic cancers. JAMA Netw. Open.

[60] Reisle, C., Davies, A. & Reisle, A. *A Platform for Oncogenomic Reporting and Interpretation, bcgsc/pori*. (2021). 10.5281/zenodo.5728141

[61] Reisle, C. *A Platform for Oncogenomic Reporting and Interpretation, bcgsc/pori_cbioportal*. (2021). 10.5281/zenodo.5730702

[62] Reisle, C., Muhammadzadeh, A. & Pellegrini, B. *A Platform for Oncogenomic Reporting and Interpretation, bcgsc/pori_graphkb_api*. (2021). 10.5281/zenodo.5730582

[63] Reisle, C., Beckie, I., Pham, D., Li, J. & Davies, A. *A Platform for Oncogenomic Reporting and Interpretation, bcgsc/pori_graphkb_client*. (2021). 10.5281/zenodo.5730456

[64] Reisle, C., Muhammadzadeh, A. & Grisdale, C. J. *A Platform for Oncogenomic Reporting and Interpretation, bcgsc/pori_graphkb_loader*. (2021). 10.5281/zenodo.5737760

[65] Reisle, C. *A Platform for Oncogenomic Reporting and Interpretation, bcgsc/pori_graphkb_parser*. (2021). 10.5281/zenodo.5730403

[66] Reisle, C. *A Platform for Oncogenomic Reporting and Interpretation, bcgsc/pori_graphkb_python*. (2021). 10.5281/zenodo.5730527

[67] Reisle, C. *A Platform for Oncogenomic Reporting and Interpretation, bcgsc/pori_graphkb_schema*. (2021). 10.5281/zenodo.5730412

[68] Pellegrini, B. et al. *A Platform for Oncogenomic Reporting and Interpretation, bcgsc/pori_ipr_api*. (2021). 10.5281/zenodo.5728334

[69] Davies, A. et al. *A Platform for Oncogenomic Reporting and Interpretation, bcgsc/pori_ipr_client*. (2021). 10.5281/zenodo.5728425

[70] Reisle, C., Bleile, D. W. & Douglas, M. *A Platform for Oncogenomic Reporting and Interpretation, bcgsc/pori_ipr_python*. (2021). 10.5281/zenodo.5730677

