# Supplementary material for: A platform for oncogenomic reporting and interpretation
Source: Nat Commun. 2022 Feb 9;13:756. doi: 10.1038/s41467-022-28348-y (PMC8828759; doi:10.1038/s41467-022-28348-y)
Supplement: Supplementary file 3 — Reporting Summary [file 41467_2022_28348_MOESM3_ESM.pdf]

nature research

Corresponding author(s): Dr. Steven Jones
Last updated by author(s): Dec 7, 2021

Reporting Summary

Nature Research wishes to improve the reproducibility of the work that we publish. This form provides structure for consistency and transparency in reporting. For further information on Nature Research policies, see our Editorial Policies and the Editorial Policy Checklist.

Statistics

For all statistical analyses, confirm that the following items are present in the figure legend, table legend, main text, or Methods section.

n/a	Confirmed	
☐	☒	The exact sample size (*n*) for each experimental group/condition, given as a discrete number and unit of measurement
☐	☒	A statement on whether measurements were taken from distinct samples or whether the same sample was measured repeatedly
☐	☒	The statistical test(s) used AND whether they are one- or two-sided<br>*Only common tests should be described solely by name; describe more complex techniques in the Methods section.*
☐	☒	A description of all covariates tested
☐	☒	A description of any assumptions or corrections, such as tests of normality and adjustment for multiple comparisons
☐	☒	A full description of the statistical parameters including central tendency (e.g. means) or other basic estimates (e.g. regression coefficient) AND variation (e.g. standard deviation) or associated estimates of uncertainty (e.g. confidence intervals)
☐	☒	For null hypothesis testing, the test statistic (e.g. *F*, *t*, *r*) with confidence intervals, effect sizes, degrees of freedom and *P* value noted<br>*Give P values as exact values whenever suitable.*
☒	☐	For Bayesian analysis, information on the choice of priors and Markov chain Monte Carlo settings
☒	☐	For hierarchical and complex designs, identification of the appropriate level for tests and full reporting of outcomes
☒	☐	Estimates of effect sizes (e.g. Cohen's *d*, Pearson's *r*), indicating how they were calculated

Our web collection on statistics for biologists contains articles on many of the points above.

Software and code

Policy information about availability of computer code

Data collection	Data was collected into a GraphKB instance via the GraphKB Loaders repository here:<br>https://github.com/bcgsc/pori_graphkb_loader (v5.0.0)
Data analysis	The platform consists of several separate repositories:<br><br>https://github.com/bcgsc/pori (v1.0.0)<br>https://github.com/bcgsc/pori_ipr_python (v2.0.4)<br>https://github.com/bcgsc/pori_graphkb_python (v1.5.1)<br>https://github.com/bcgsc/pori_graphkb_client (v4.2.3)<br>https://github.com/bcgsc/pori_graphkb_api (v3.13.4)<br>https://github.com/bcgsc/pori_ipr_client (v6.6.3)<br>https://github.com/bcgsc/pori_ipr_api (v7.2.1)<br>https://github.com/bcgsc/pori_graphkb_schema (v3.15.1)<br>https://github.com/bcgsc/pori_graphkb_parser (v1.1.3)<br>https://github.com/bcgsc/pori_cbioportal (v0.1.1)

For manuscripts utilizing custom algorithms or software that are central to the research but not yet described in published literature, software must be made available to editors and reviewers. We strongly encourage code deposition in a community repository (e.g. GitHub). See the Nature Research guidelines for submitting code & software for further information.

Data

Policy information about availability of data

All manuscripts must include a data availability statement. This statement should provide the following information, where applicable:

- Accession codes, unique identifiers, or web links for publicly available datasets
- A list of figures that have associated raw data
- A description of any restrictions on data availability

The Disease Ontology data used in this study are available from the github repository (v2020-06-18) [https://github.com/DiseaseOntology/HumanDiseaseOntology/blob/v2020-06-18/src/ontology/releases/2020-06-18/doid.json]. The FDA SRS data used in this study are available from the FDA downloads page (March 27 2020 release) [https://fdasis.nlm.nih.gov/srs/download/srs/UNIIs_20200327.zip]. The DrugBank data used in this study are available from the DrugBank releases page (v5.1.8) [https://go.drugbank.com/releases/5-1-8/downloads/all-full-database]. The NCIt data used in this study are available from the NCIt ftp downloads page (20.06e) [https://evs.nci.nih.gov/ftp1/NCI_Thesaurus/archive/2020/20.06e_Release/Thesaurus.FLAT.zip]. The ChEMBL data used in this study are available from the ChEMBL FTP downloads page [http://doi.org/10.6019/CHEMBL.database.27]. The OncoTree data used in this study are available from the OncoTree API (oncotree_2020_04_01) [http://oncotree.mskcc.org/api/tumorTypes?version=oncotree_2020_04_01]. The clinical trials data used in this study are available from ClinicalTrials.gov [https://clinicaltrials.gov/AllPublicXML.zip]. The CIViC data used in this study is available from the CIViC API [https://civicdb.org/api]. The OncoKB data are available under restricted access due to licensing requirements, access can be obtained by registering for a license of the OncoKB data [https://www.oncokb.org]. The CGI data used in this study is available from the CGI webpage [https://www.cancergenomeinterpreter.org/data/cgi_biomarkers_latest.zip]. The COSMIC data used in this study is available from the file downloads page of the COSMIC website (v92) [https://cancer.sanger.ac.uk/cosmic/file_download/GRCh38/cosmic/v92/CosmicResistanceMutations.tsv.gz]. The DoCM data used in this study is available from the DoCM API [http://docm.info/api/v1/variants]. TCGA PanCancer Atlas Studies data was accessed and downloaded from cBioportal.org [http://www.cbioportal.org/datasets]. A full list of sample accession numbers is provided in Supplementary Data 1. The Genomic and transcriptomic datasets for the cholangiocarcinoma case study have been previously deposited and are available in the European Genome-phenome Archive under accession number EGAD00001002623 [https://ega-archive.org/datasets/EGAD00001002623]. The report for this data is available via the PORI demo (0bdec40b-04d7-4264-aa3f-7ddb4cbeebf5) [https://bcgsc.github.io/pori/demo]. The GTEx datasets used for the cholangiocarcinoma case analyses described in this manuscript were obtained from dbGaP through accession number phs000424.v6.p1 [https://www.ncbi.nlm.nih.gov/projects/gap/cgi-bin/study.cgi?study_id=phs000424.v6.p1] and TCGA data for this case were derived from RNA-Seq gene expression data now available through the Genomic Data Commons Data Portal (https://portal.gdc.cancer.gov/), project names starting with "TCGA-".The Illumina human body map (2.0) data used for this case is available from the Gene Expression Omnibus under accession number GSE30611 [https://www.ncbi.nlm.nih.gov/geo/query/acc.cgi?acc=GSE30611]. Source data are provided with this paper.

Field-specific reporting

Please select the one below that is the best fit for your research. If you are not sure, read the appropriate sections before making your selection.

☒ Life sciences ☐ Behavioural & social sciences ☐ Ecological, evolutionary & environmental sciences

For a reference copy of the document with all sections, see nature.com/documents/nr-reporting-summary-flat.pdf

Life sciences study design

All studies must disclose on these points even when the disclosure is negative.

Sample size	The sample size of the clinical reports reported in the manuscript was determined as to showcase some of the features of the software in how it presented the relevant clinical information in the report. We chose to analyze all samples (excluding the LUAD cohort, see methods) from the pan-cancer genome atlas project (n=9,961) as it represents a large, well-known, set of samples with multiple variant types. This was done for demonstration on the process of the software and no statistical inferences were made from the output.
Data exclusions	Data is omitted from the reports if it is considered not relevant for clinical decision making at the current time.
Replication	Our software produces identical outputs from a given input. The output is therefore deterministic and we have tested the software to demonstrate this. In each component of the platform we have included unit and integration tests to assure replicability. These are run on every change to the software and the results are available publicly with the github repository. We confirm that all attempts at replication were successful.
Randomization	The input of samples into our software is not randomized but specifically determined by the user. Randomization is not relevant to our analysis as the output is deterministic and independent of the order in which samples are processed.
Blinding	In the process of developing the software, samples were not blinded. The development of the software was to faithfully record and report clinically relevant information provided in larger genomic analysis outputs. Therefore blinding of information would not have improved this process.

Reporting for specific materials, systems and methods

We require information from authors about some types of materials, experimental systems and methods used in many studies. Here, indicate whether each material, system or method listed is relevant to your study. If you are not sure if a list item applies to your research, read the appropriate section before selecting a response.

Materials & experimental systems

n/a	Involved in the study
☒	☐ Antibodies
☒	☐ Eukaryotic cell lines
☒	☐ Palaeontology and archaeology
☒	☐ Animals and other organisms
☒	☐ Human research participants
☒	☐ Clinical data
☒	☐ Dual use research of concern

Methods

n/a	Involved in the study
☒	☐ ChIP-seq
☒	☐ Flow cytometry
☒	☐ MRI-based neuroimaging
